# Expanded Endoscopic Endonasal Approach and Odontoidectomy for Atlantoaxial Synovial Cyst

**DOI:** 10.1055/a-2852-7513

**Published:** 2026-07-21

**Authors:** Paarth Patel, Jonathan Espinosa, Thomas Tyler Patterson, Erin Lopez, Cristian Gragnaniello

**Affiliations:** 1 Department of Neurosurgery 12345 UT Health San Antonio Long School of MedicineSan AntonioTexasUnited States; 2 Department of Neurosurgery 14742 UT Health San AntonioSan AntonioTexasUnited States; 3 Department of Otolaryngology—Head and Neck Surgery 14742 UT Health San AntonioSan AntonioTexasUnited States

**Keywords:** atlantoaxial joint, synovial cyst, retro-odontoid, odontoidectomy, transnasal, transoral, endoscopic endonasal approach

## Abstract

**Introduction** Synovial cysts of the atlantoaxial joint (AAJ) are rare lesions that may cause progressive spinal cord compression and cervical myelopathy. Surgical management of retro-odontoid pathology is challenging due to complex regional anatomy and proximity to critical neurovascular structures. Traditional approaches, including transoral and posterior transdural techniques, are associated with notable morbidity. The endoscopic endonasal approach (EEA) provides a minimally invasive alternative with direct access to the retro-odontoid space. We report an isolated AAJ synovial cyst treated with an endoscopic endonasal odontoidectomy as the sole anterior approach, highlighting technical refinements that optimized access to the retro-odontoid space.

**Case Description** A 48-year-old woman with a history of C4 corpectomy and C3 to C7 anterior fusion presented with progressive neck pain and cervical myelopathy, secondary to a retro-odontoid cystic lesion causing spinal cord compression. She underwent an endoscopic endonasal odontoidectomy and lesion resection followed by posterior occipitocervical fusion. Surgical exposure was optimized through posterior septectomy and targeted drilling of the odontoid process to access the retro-odontoid space. Excision and adequate decompression of the spinal cord were achieved, and histopathology was consistent with synovial cyst.

**Conclusion** The EEA allowed effective ventral decompression of the AAJ synovial cyst while minimizing morbidity associated with transoral and posterior transdural approaches. Strategic modifications to improve surgical angulation and maneuverability can overcome traditional limitations of the endonasal corridor. This approach represents a viable, minimally invasive option for select retro-odontoid pathologies while reducing trauma to oropharyngeal tissue and avoiding dural violation at the AAJ.

## Introduction

Retro-odontoid lesions pose unique surgical challenges due to dense neurovascular and osseous anatomy of the region as well as proximity of critical structures that complicate surgical access. The transoral approach has traditionally been used to treat retro-odontoid pathology but is associated with significant morbidity related to disruption of the oral cavity and pharyngeal musculature. 1 2 A posterior transdural approach has been recently introduced involving laminectomies and dural incisions to access the odontoid. 3 While this technique avoids oropharyngeal damage, it introduces risks inherent to dural violation including cerebrospinal fluid (CSF) leak. An alternative anterior approach is the endoscopic endonasal approach (EEA), offering a minimally invasive strategy with adequate visualization while mitigating many morbidities associated with other approaches. 4

Synovial cysts are benign, fluid-filled dilations of the synovial lining that arise from joint capsules. 5 They occur most commonly in the lumbar spine, with decreasing prevalence in the thoracic and cervical regions, and are rare at the atlantoaxial joint (AAJ). 6 7 8 Bruder et al identified 3041 cases, with 193 (6.3%) in the cervical region and only 32 (1.1%) involved the C1 to C2 joint. 9 As upper cervical synovial cysts enlarge, they may compress the spinal cord or adjacent nerve roots, leading to worsening myeloradiculopathy, necessitating surgical intervention. 10 Symptoms can progress over months, and advanced cases may involve os odontoideum, vertebral artery damage, brainstem compression, or cranial nerve involvement. 9 11 12 13 14

To our knowledge, excision of an isolated AAJ synovial cyst using an endoscopic endonasal odontoidectomy as the sole anterior approach has not been previously reported. We describe a novel expanded transnasal odontoidectomy that incorporates posterior septectomy, palatal drilling, and dynamic soft-palate retraction to achieve access to the retro-odontoid space.

## Case Description



**Video 1** Endoscopic endonasal intraoperative footage illustrating the approach from the nasopharynx through the anterior arch of C1 to the synovial cyst occupying the retro-odontoid space. The cyst is visualized overlying the anterior cervical dura, and the sequence captures the start of its endoscopic excision.


**Video 2** Intraoperative endoscopic endonasal surgical footage depicting the complete operative corridor from the nasal cavity through the nasopharynx, anterior arch of C1, and odontoid process to the anterior dura after the synovial cyst excision. This sequence highlights the minimally invasive trajectory achieved with the endonasal approach for access to the retro-odontoid space.


**Fig. 1:**
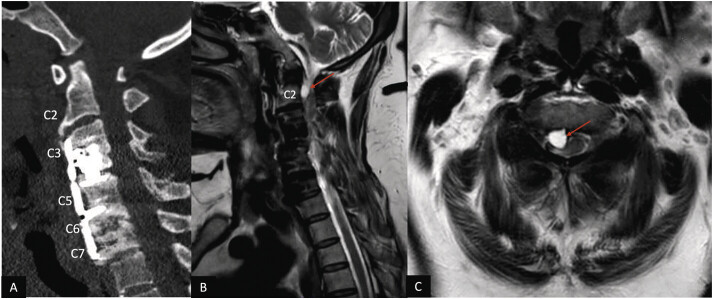
Preoperative sagittal CT scan ( **A** ) demonstrating prior anterior cervical instrumentation from C3 to C7, including a C4 corpectomy reconstructed with an interbody cage and anterior plate fixation. Preoperative sagittal ( **B** ) and axial ( **C** ) T2-weighted MRI demonstrating a retro-odontoid synovial cyst (arrow) compressing the cervical spinal cord at the level of C2. CT, computed tomography; MRI, magnetic resonance imaging.

A 48-year-old woman with a history of C3 to C7 anterior fusion with C4 corpectomy presented with progressive neck pain, upper extremity radiculopathy, and gait instability ( Fig. 1 ). Magnetic resonance imaging (MRI) demonstrated a retro-odontoid cystic lesion causing spinal cord compression with associated T2 signal change ( Fig. 1 ). Given the progressive neurological symptoms and interval enlargement of the lesion on serial imaging, surgical intervention was recommended. After discussion, the patient consented to a combined anterior–posterior decompression and fusion with an EEA to the dens for lesion resection.

**Fig. 2:**
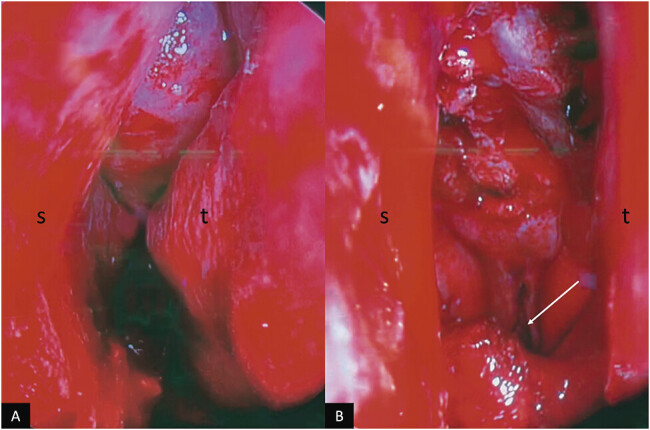
Endoscopic view of the anterior nasal cavity ( **A** ), showing a nasal turbinate (t) along the lateral wall and the nasal septum (s) along the medial wall. Endoscopic view of the posterior nasal cavity ( **B** ), demonstrating the nasopharynx with the posterior nasopharyngeal incision (arrow).

**Fig. 3:**
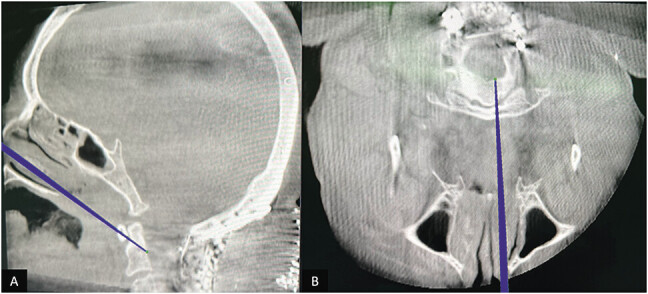
Intraoperative sagittal ( **A** ) and axial ( **B** ) neuronavigation images depicting the endonasal surgical pathway extending from the nasal cavity through the nasopharynx to the odontoid process.

**Fig. 4:**
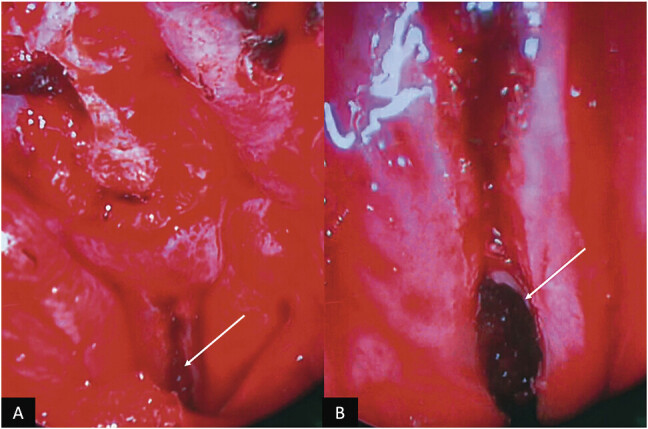
Endoscopic view of the posterior nasopharyngeal incision (arrow), showing the surrounding nasopharynx ( **A** ) and the open incision ( **B** ).

**Fig. 5:**
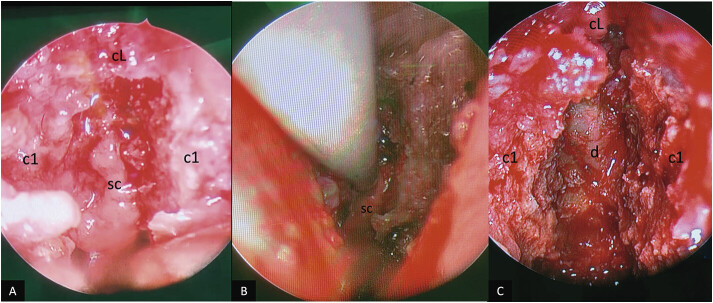
Endoscopic view of the retro-odontoid space before ( **A** ), during ( **B** ) and after ( **C** ) excision of the synovial cyst. The clivus (cL), drilled portions of the anterior arch of C1 (c1), synovial cyst (sc), and anterior dura (d) after synovial cyst excision are labeled.

The posterior occipitocervical fusion was performed first to address anticipated instability after resection of the anterior arch, odontoid process, and associated stabilizing ligaments. This portion was uncomplicated. Next, the EEA was initiated. The bilateral inferior turbinates were outfractured to widen the nasal corridor ( Fig. 2 ). A posterior septectomy was completed using needle-tip monopolar cautery to incise the mucosa with through-cutting instruments utilized to remove the vomer to the level of the palatine bone. A high-speed drill was then used to reduce the midline palatine bone, improving inferior transnasal angulation toward the cervical spine. For soft-palate management, the uvula was secured with two passes of a 2–0 silk suture in a double-loop configuration to distribute tension and prevent tissue injury. The suture was placed transorally and controlled with a hemostat, allowing manually adjustable palatal retraction throughout the procedure. The posterior pharyngeal wall was inspected, and the locations of C1, C2, and the clivus were confirmed using image guidance ( Fig. 3 ). A midline incision was made in the posterior pharyngeal wall using extended needle-tip monopolar cautery, and the wound edges were coagulated with bipolar radiofrequency cautery ( Fig. 4 ). Excess soft tissue was debrided with a microdebrider. Dissection was carried down to bone, at which point the periosteum was incised using monopolar cautery. Once the inferior clivus, anterior arch of C1, and a portion of the C2 vertebral body were exposed, a high-speed drill with matchstick and diamond burrs was used to thin the anterior arch of C1. Punch instruments were then used to complete resection of the C1 anterior arch over a width of approximately 1 to 1.5 cm, with meticulous care taken to avoid the medial aspects of the C1 lateral masses. The odontoid process was subsequently freed from the apical and alar ligaments, and a high-speed drill was used to remove the dens in a cranial-to-caudal fashion. The posterior cortical wall of the dens was elevated using curettes and punch instruments, allowing entry into the retro-odontoid space. Upon traversing the lesion capsule, cystic fluid was encountered. The lesion was then carefully excised ( Fig. 5 , Video 1 ). A portion was submitted for histopathologic analysis, confirming the diagnosis of synovial cyst. No intraoperative CSF leak was observed. Once adequate spinal cord decompression was achieved, closure was performed ( Video 2 ). Gentamicin-soaked absorbable gelatin sponges were placed within the surgical bed, followed by absorbable nasal packing. A nasogastric tube was then inserted under direct endoscopic visualization to ensure appropriate passage without violation of the nasopharyngeal incision. Subsequently, a fingercot comprised of size 7 blue glove containing nasal sponge packing secured with 2–0 silk suture was placed in the contralateral nostril and positioned against the nasopharyngeal wound to maintain packing stability. The attached silk suture was taped to the nasal dorsum.

**Fig. 6:**
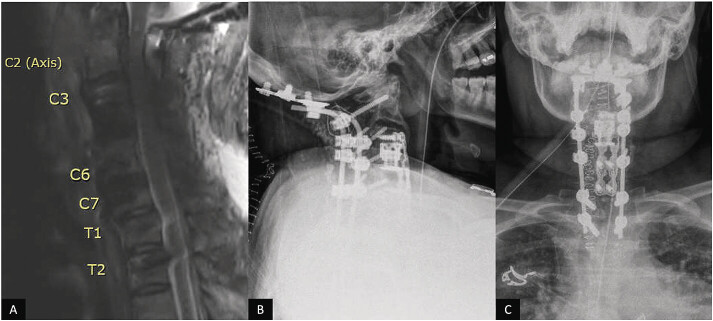
Postoperative T2-weighted MRI ( **A** ) demonstrating excision of the retro-odontoid synovial cyst. Postoperative sagittal ( **B** ) and anteroposterior ( **C** ) plain radiographs demonstrating stable posterior occipitocervical fusion with appropriate alignment and hardware positioning. MRI, magnetic resonance imaging.

**Fig. 7:**
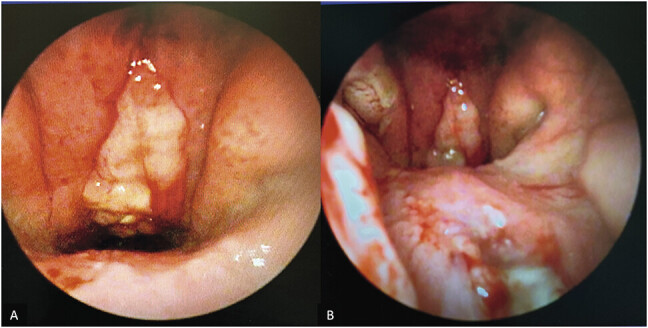
One-month postoperative endoscopic image showing progressive mucosal healing at the nasopharyngeal incision site. The zoomed-in image ( **A** ) highlights a well-healing wound, and the wider view ( **B** ) provides context within the nasal cavity.

The patient had an uneventful postoperative course. There was no evidence of velopharyngeal insufficiency, and the patient tolerated a full diet. Swallowing function remained intact, and no dietary restrictions were required. Immediate postoperative MRI demonstrated excision of the synovial cyst, and postoperative plain radiographs confirmed stable arthrodesis without hardware-related complications ( Fig. 6 ). Endoscopic evaluation of the nasopharyngeal mucosa at 1-month follow-up demonstrated appropriate healing ( Fig. 7 ). At 3- and 6-month follow-up, the patient reported significant improvement in hand numbness with no residual myelopathic or gait symptoms.

## Discussion

Due to its wide exposure of the dens and adjacent abnormal tissue, the transoral approach has traditionally been regarded as the gold standard for managing retro-odontoid pathology. 15 16 17 However, this approach necessitates extensive disruption of the pharynx, including division of the soft palate, and is associated with substantial morbidity such as dysphagia, speech disturbances, and respiratory complications. 2 Additional complications include arterial injuries, pneumonia, pharyngeal wound dehiscence, and even the need for a tracheostomy. 18 In contrast, the EEA preserves the integrity of the oropharynx, maintaining baseline swallowing function, and can avoid many of these permanent, life-altering morbidities. Komotar et al found that a transnasal approach significantly accelerated extubation and oral feeding by approximately 3 days (0.33 vs. 3.58) and 1.5 days (3.86 vs. 5.24), respectively, compared with a transoral approach, likely reflecting traversal of the nasopharynx rather than the oropharynx and a smaller pharyngeal incision. 19 Ponce-Gomez et al reported a reduction in postoperative stay by 3.7 days (2.8 vs. 6.5) after an EEA, likely attributed to faster extubation and return to oral intake. 20 These findings suggest that the EEA facilitates a faster and less complicated recovery, with potential reductions in patient morbidity and hospital costs.

Alternative posterior and lateral approaches to retro-odontoid pathology have also been described. Posterior transdural techniques preserve the pharynx and odontoid but require multiple dural incisions, exposing critical intradural structures and increasing the risk of CSF-related complications. 21 22 Jamshidi et al described sectioning the dentate ligament, partial sacrifice of the C2 nerve root, and mobilization of the 11th cranial nerve, with one postoperative pseudomeningocele occurring among eight cases. 22 Lateral cervical approaches provide another alternative but may increase the risk of vertebral artery injury and often necessitate C2 nerve root resection. 23 In contrast, the EEA preserves these key neurovascular structures that limit exposure to posterior and lateral approaches, minimizing the risk of certain permanent neurological deficits.

The EEA is an established and effective technique for a variety of odontoid and retro-odontoid pathologies including rheumatoid pannus, neoplasms, pseudotumors, basilar invagination, os odontoideum, and platybasia. 4 16 24 Prior reports have described endonasal approaches for cystic lesions involving the craniovertebral junction. 25 In contrast, the present case describes a synovial cyst confined to the AAJ, necessitating targeted inferior access directly to the retro-odontoid space at the junction of C1 and C2. Although the transnasal corridor may be perceived as limited in working angles and instrument maneuverability, several technical modifications can improve exposure. In the present case, resection of portions of the vomer and midline hard palate combined with controlled soft-palate retraction enhanced inferior angulation toward the dens. Rather than nasogastric tubing passed transnasally and exiting the oral cavity to displace the soft palate, inferior visualization was optimized using a double-looped uvular suture that allowed dynamic, intraoperative control. While soft-palate or uvular retraction has been described in prior reports, this technique represents a practical variation that avoids nasal tubing and permits adjustable retraction throughout the procedure. 26 27 Additionally, limited clival resection, if necessary, may enhance superior exposure for conditions such as basilar invagination. Together these refinements help overcome traditional limitations to the EEA, broadening its applicability to a range of surgical objectives.

## Conclusion

The EEA offers a minimally invasive alternative for excision of AAJ synovial cysts, preserving critical neurovascular structures while reducing morbidity associated with transoral and transdural approaches. Strategic optimization of surgical angulation through targeted bony modification and soft-tissue retraction can overcome traditional limitations of the endonasal corridor and enable effective ventral decompression. Furthermore, by avoiding extensive oropharyngeal disruption, this approach may facilitate improved postoperative recovery. This case highlights the versatility of the EEA for isolated retro-odontoid pathology and supports its consideration as a viable anterior approach.
